# Isolation and Characterization of Fengycins Produced by *Bacillus amyloliquefaciens* JFL21 and Its Broad-Spectrum Antimicrobial Potential Against Multidrug-Resistant Foodborne Pathogens

**DOI:** 10.3389/fmicb.2020.579621

**Published:** 2020-12-18

**Authors:** Long-Zhen Lin, Qian-Wang Zheng, Tao Wei, Zi-Qian Zhang, Chao-Fan Zhao, Han Zhong, Qing-Yuan Xu, Jun-Fang Lin, Li-Qiong Guo

**Affiliations:** ^1^Department of Bioengineering, College of Food Science, South China Agricultural University, Guangzhou, China; ^2^Research Center for Micro-Ecological Agent Engineering and Technology of Guangdong Province, Guangzhou, China

**Keywords:** fengycin, *Bacillus amyloliquefaciens*, antimicrobial activity, multidrug-resistant foodborne pathogens, probiotics, lipopeptides

## Abstract

The continuing emergence and development of pathogenic microorganisms that are resistant to antibiotics constitute an increasing global concern, and the effort in new antimicrobials discovery will remain relevant until a lasting solution is found. A new bacterial strain, designated JFL21, was isolated from seafood and identified as *B. amyloliquefaciens*. The antimicrobial substance produced by *B. amyloliquefaciens* JFL21 showed low toxicity to most probiotics but exhibited strong antimicrobial activities against multidrug-resistant foodborne pathogens. The partially purified antimicrobial substance, Anti-JFL21, was characterized to be a multiple lipopeptides mixture comprising the families of surfactin, fengycin, and iturin. Compared with commercially available polymyxin B and Nisin, Anti-JFL21 not only could exhibit a wider and stronger antibacterial activity toward Gram-positive pathogens but also inhibit the growth of a majority of fungal pathogens. After further separation through gel filtration chromatography (GFC), the family of surfactin, fengycin, and iturin were obtained, respectively. The results of the antimicrobial test pointed out that only fengycin family presented marked antimicrobial properties against the indicators of *L. monocytogenes*, *A. hydrophila*, and *C. gloeosporioides*, which demonstrated that fengycins might play a major role in the antibacterial and antifungal activity of Anti-JFL21. Additionally, the current study also showed that the fengycins produced by *B. amyloliquefaciens* JFL21 not only maintained stable anti-*Listeria* activity over a broad pH and temperature range, but also remained active after treatment with ultraviolet sterilization, chemical reagents, and proteolytic enzymes. Therefore, the results of this study suggest the new strain and its antimicrobials are potentially useful in food preservation for the biological control of the multidrug-resistant foodborne pathogens.

## Introduction

Foodborne diseases have been a serious global public health issue and most of them are caused by foodborne pathogens such as *Salmonella*, *Shigella*, *Vibrio*, *Escherichia coli* O157, *Yersinia enterocolitica*, *Listeria monocytogenes*, *Staphylococcus aureus*, and *Aspergillus flavus*, etc. (World Health Organization, 2015; European Food Safety Authority, 2018; Tack et al., 2019). According to the data reported by the World Health Organization (WHO), foodborne diseases have caused approximate 600 million illness and 420, 000 deaths in the world and at least $100 billion costs in low- and middle-income countries every year, and the actual number of cases is also likely to be underestimated due to the high failure rate of reporting foodborne diseases worldwide (World Health Organization, 2015, 2019). Although antibiotics are essential to treat infections caused by foodborne pathogens, their overuse and misuse has been linked to the emergence and spread of multiple resistant strains during the past decades and has led to public awareness (Garedew et al., 2015; Kuch et al., 2018; U.S. Centers for Disease Control and Prevention, 2019). According to the government data of U.S. Centers for Disease Control and Prevention (CDC), antibiotic-resistant infections from foodborne germs (bacteria and fungi) still cause more than 2.8 million illnesses and 35,000 deaths in the United States each year despite attempts to combat the problem (U.S. Centers for Disease Control and Prevention, 2019). Thus, there is an urgent need for alternatives to antibiotics to fight against the foodborne pathogens.

In recent years, many studies have shown that the cyclic lipopeptides (CLPs) produced by *Bacillus* spp. have potent antimicrobial activity against antibiotic-resistant strains and can be generally divided into three main families: surfactin, fengycin, and iturin (Chi et al., 2015; Jemil et al., 2017; Perez et al., 2017; Piewngam et al., 2018). Due to their specific amphiphilic structure, CLPs primarily destroy target organisms by directly disrupting the integrity of the plasma membrane or cell wall in a detergent-like manner, and thus display a lower propensity to develop resistance than do conventional antibiotics (Banat et al., 2010; Mandal et al., 2013; Singh and Abraham, 2014; Patel et al., 2015; Ndlovu et al., 2017). Besides, CLPs are biodegradable, biocompatible, eco-friendly, relatively non-toxic, and resistant to extreme conditions of temperatures, pH, and salinity (Pradhan et al., 2013; Ben Ayed et al., 2017). Because of these attractive characteristics, naturally produced antimicrobial CLPs have received increasing attention as promising new antibiotic candidates for food, pharmaceutical, and biomedical applications.

Considering the increasingly prominent problems of antibiotic contamination and food safety, this research aimed to search for candidate *Bacillus* strains that could produce a potent antimicrobial agent to combat various foodborne pathogens with multi-drug resistant profiles. Besides, FITR, HPLC, and MALDI-TOF MS analysis were exploited to identify the structural characteristics of the partially purified antimicrobial substances Anti-JFL21. Moreover, the different lipopeptide families in Anti-JFL21 was further separated, and the effective antimicrobial family was elucidated. what’s more, the fengycins stability after the treatment of heating, pH change, ultraviolet sterilization, enzymes, and chemical reagents was determined to evaluate the possible incorporation in the food production chain.

## Materials and Methods

### Microorganisms and Cultivation Conditions

In this study, the three *Bacillus* isolates named JFL21, LQG17, and LQG36, with excellent antimicrobial properties were, respectively, isolated from the gut of hairtail, fermented soybean, and pickle purchased from Guangzhou farmers market. *Bacillus subtilis* 168, one of the model organisms of the *Bacillus* genus, was purchased from the Bacillus Genetic Stock Center (BGSC). The thirty-three indicator strains used for evaluation of antibacterial and antifungal activity were selected for their importance as probiotics or pathogens in food and seafood products. The source and culture conditions of the thirty-three indicator strains are listed in Table 1. The bacterial or fungal cultures were preserved in 25% glycerol at −80°C.

**TABLE 1:** Drug resistance of representative bacterial pathogens, pathogenic fungi, and probiotics in food and seafood products.^*a*^

Indicator microorganisms	Source^*b*^	Media^*c*^	T(°C)	Antibiotic sensitivity^*d*^											
				1	2	3	4	5	6	7	8	9	10	11	12
**Lactic acid bacteria (Probiotics)**															
*Lactobacillus plantarum*	Yogurt	MRS	37	+++	++	−	−	+++	−	+++	−	+++	−	−	−
*Pediococcus pentosaceus*	Tilapia gut	MRS	37	++	++	−	−	+++	−	+++	−	++	−	−	−
*Lactobacillus casei*	Pickle	MRS	37	+++	−	−	+++	−	−	−	−	++	−	−	−
*Lactococcus lactis*	*Pleurotus eryngii*	MRS	37	+++	++	+++	++	+++	++	+++	−	++	−	−	−
*Leuconostoc mesenteroides*	Shrimp gut	MRS	37	+++	+++	+++	+++	+++	++	+++	++	+++	−	−	−
**Gram-positive pathogen**															
*Listeria monocytogenes*	ATCC 19111	BHI	37	+++	−	+++	−	++	−	++	++	+++	−	−	−
*Staphylococcus aureus*	ATCC 12600	TSA	37	+++	++	+++	++	+++	+++	++	++	+++	−	−	−
*Staphylococcus epidermidis*	ATCC 14990	BHI	37	−	++	++	−	−	+	+	+	+++	+	−	−
*Staphylococcus warneri*	ATCC 27836	TSA	37	−	−	+++	+	−	++	−	−	++	−	−	−
*Staphylococcus haemolyticus*	ATCC 29970	TSA	37	+++	−	+++	+++	−	++	−	−	+++	−	−	−
*Bacillus cereus*	ATCC 14579	TSA	37	+	++	+	+	++	++	+	+++	+++	−	−	−
**Gram-negative pathogen**															
*Vibrio parahaemolyticus*	ATCC 17802	BHI	37	++	−	−	−	−	+	−	+	++	−	−	−
*Vibrio harveyi*	ATCC 33843	TSA	37	+++	−	++	−	−	−	+	+++	+++	−	−	−
*Vibrio vulnificus*	ATCC 27562	BHI	37	+++	−	−	−	−	+	+++	+	+	−	−	−
*Vibrio campbellii*	ATCC 33863	TSA	37	−	++	+++	−	−	+	−	−	++	−	−	−
*Pseudomonas aeruginosa*	ATCC 10145	TSA	37	−	−	+++	−	−	−	−	−	+++	−	−	−
*Aeromonas hydrophila*	ATCC 7966	TSA	37	−	+++	+++	++	−	++	+	−	++	−	−	−

**Indicator microorganisms**	**Source^*b*^**	**Media^*c*^**	**T(°C)**	**Antibiotic sensitivity^*d*^**											
				1	2	3	4	5	6	7	8	9	10	11	12

*Escherichia coli* O157:H7	ATCC 35150	TSA	37	+++	+	++	−	−	++	+	−	++	−	−	−
*Salmonella choleraesuis*	ATCC 10708	TSA	37	+	+++	++	−	−	+	−	+	+	−	−	−
*Salmonella typhimurium*	CMCC(B) 50115	TSA	37	+++	−	+++	−	−	++	−	+	+	−	−	−
*Shigella flexneri*	ATCC 29903	TSA	37	+++	+++	+++	−	−	+	++	−	+	−	−	−
*Yersinia enterocolitica*	ATCC 9610	TSA	37	−	−	−	−	−	++	+	++	+++	−	−	−
*Proteus mirabilis*	ATCC 29906	TSA	37	+++	+	−	−	−	+	−	++	−	−	−	−
*Cronobacter sakazakii*	ATCC 51329	TSA	37	+++	++	+	−	−	++	+	+	++	+	−	−
*Klebsiella pneumoniae*	ATCC 13883	TSA	37	−	+++	+++	++	++	++	++	−	++	−	−	−
*Enterobacter aerogenes*	CMCC(B) 45103	TSA	37	−	−	+++	−	−	+	−	−	++	−	−	−
**Pathogenic fungi**															
*Rhizopus oryzae*	GIM 3.126	PDA	28	−	−	−	−	−	−	−	−	−	−	−	−
*Aspergillus niger*	Grape	PDA	28	−	−	−	−	−	−	−	−	−	−	−	++
*Aspergillus flavus*	GIM 3.18	PDA	28	−	−	−	−	−	−	−	−	−	−	−	−
*Aspergillus fumigatus*	GIM 3.19	PDA	28	−	−	−	−	−	−	−	−	−	−	+++	−
*Peronophythora litchii*	Litchi	PDA	28	−	−	−	−	−	−	−	−	−	−	−	++
*Colletotrichum gloeosporioides*	Mango	PDA	28	−	−	−	−	−	−	−	−	−	−	−	−
*Penicillium polonicum*	Orange	PDA	28	−	−	−	−	−	−	−	−	−	−	+++	−

*^*a*^The experimental concentration of each antibiotic was 100 ug/ml. 1, Ampicillin; 2, chloramphenicol; 3, Tetracycline; 4, Chlorotetracycline; 5, Erythromycin; 6, kanamycin; 7, Azithromycin; 8, Apramycin; 9, Gentamicin; 10, Spectinomycin; 11, Benomyl; 12, Actidione. ^*b*^ATCC, American Type Culture Collection; CMCC, National Center for Medical Culture Collections; *Rhizopus oryzae, Aspergillus flavus*, and *Aspergillus fumigatus* were purchased from Guangdong Culture Collection Center; The other indicator bacteria were isolated from different food samples and stored in our laboratory. ^*c*^MRS, de Man, Rogosa and Sharpe medium; TSA, Trypticase Soya Agar; BHI, Brain Heart Infusion; PDA, Potato Dextrose Agar. ^*d*^The inhibition zone (IZ) were measured based on the agar disk diffusion assay. −: Sensitivity, IZ ≤ 9; +: Slight resistance, 9 ≤ IZ < 15 mm; ++: Moderate resistance, 15 ≤ IZ < 21 mm; +++: High resistance, IZ ≥ 21 mm.*

### Measurement of Drug Resistance of Probiotics and Pathogens

Drug resistance of probiotics and pathogens were determined according to the agar disk diffusion assay (Xu et al., 2014; Perez et al., 2017). Briefly, Sterile Oxford cups (10 mm × 6 mm × 8 mm, height × inner diameter × outer diameter) were placed on the assay medium seeded with the fresh culture suspension of different indicator strains (about 10^8^ colony forming units/mL for bacterial cells and 10^6^ spores/mL for fungal strains). Each cup was added with 100 μL of commonly used antibiotics (100 μg/mL) (Table 1). Besides, the same amount of sterile water, anhydrous ethanol, DMSO, and 0.01 mol/L HCl that applied to prepare antibiotics were used as the negative control to subtract the inhibitory activity of the solvents. After incubation 24 h at 37°C for bacteria and 7 days at 28°C for fungal strains, the inhibition zones were measured and recorded as a mean diameter (mm). All tests were conducted in triplicate.

### Identification of Bacterial Strain and Phylogenetic Analysis

Genomic DNA from the *Bacillus* spp. was extracted using the HiPure Bacterial DNA Kit (Magen, China) according to the manufacturer’s protocol. The 16S rRNA gene fragment from each of the isolates was amplified using universal primers 27F (5′-AGAGTTTGATCCTGGCTCAG-3′) and 1492R (5′-TACGGTTACCTTGTTACGACTT-3′) according to the method described by Dimkić et al. (2017). The amplified product was purified with the QIAquick PCR purification kit (Qiagen, Germany) and sent for sequencing to Tianyi Huiyuan Bioscience & Technology Inc. (Guangzhou, China). The sequences obtained were deposited in GenBank under the accession numbers MT159453 for *Bacillus* sp. JFL21, MT159454 for *Bacillus* sp. LQG17, and MT159455 for *Bacillus* sp. LQG36. The homology comparison of the obtained sequences with previously sequenced genes in the GenBank database was performed, using the National Center for Biotechnology Information’s Blast search program (Bethesda, United States). The most closely related sequences of strain types were aligned using Clustal W software, and phylogenetic trees were constructed in MEGA version 7 using the Neighbor-joining method with 1,000 bootstrap repetitions (Dimkić et al., 2017; Perez et al., 2017).

### Production of Antimicrobial Compounds

An inoculum of the glycerol stock of each of the *Bacillus* strains was streaked onto a Luria-Bertani (LB) plate which was incubated for 24 h at 37°C. A single colony was then inoculated into LB broth medium at 30°C and 200 r/min for 16 h (approximately 5 × 10^7^ CFU/mL) to prepare seed cultures. A 1% (v/v) of inoculum was transferred into a 1 L shake flask containing 400 ml of Landy medium which was composed of the following: glucose 20 g/L, yeast extract 1 g/L, L-glutamic acid 5 g/L, KCl 0.5 g/L, MgSO_4_ 0.5 g/L, KH_2_PO_4_ 1 g/L, L-phenylalanine 2 mg/L, MnSO_4_ 5 mg/L, FeSO_4_ 0.15 mg/L, CuSO_4_ 0.16 mg/L. The initial pH was adjusted to 7.0 and submerged fermentation was carried out in the rotary shaker at 30°C and 200 r/min for 48 h to produce the antimicrobial substance. After fermentation, the culture was centrifuged at 10,000 × *g* for 15 min at 4°C to remove microbial cells. The cell-free supernatants (CFS) was further sterilized by filtration through a cellulose filter with a pore size of 0.45 μm. Thereafter the filtered CFS (100 μL) from different *Bacillus* strains was tested for antimicrobial activity against thirty-three indicator strains (Table 1) using the agar disk diffusion assay mentioned above. The same amount of uninoculated Landy medium was used as negative control.

### Extraction and Partial Purification of the Antimicrobial Compounds

The partial purification of the antimicrobial substance was carried out by a combination of acid precipitation and methanol extraction method (Sharma et al., 2015; Ndlovu et al., 2017). Briefly, the filtered CFS was adjusted to pH 2.0 by the addition of 6N HCl and allowed to precipitate at 4°C overnight. The acid precipitate was then harvested by centrifugation at 10,000 × *g* for 15 min at 4°C, and the pellet was washed twice with the 100 mL pH 2.0 distilled water (prepared through a MilliQ system from Millipore, Billerica, United States). This pellet was then resuspended in distilled water by raising the pH to 7.0, lyophilized, and solvent-extracted with methanol. The methanol-soluble fraction was dried using a rotary vacuum evaporator at 45°C. After the removal of methanol, a minimum quantity of methanol was added to dissolve the antimicrobial compound, which was again lyophilized and weighed for quantification. The crude extract from the CFS of *Bacillus* sp. JFL21 and *Bacillus* sp. LQG17 was designated as Anti-JFL21 and Anti-LQG17, respectively.

### Determination of the Antimicrobial Activity of Anti-JFL21 and Anti-LQG17

Anti-JFL21 and Anti-LQG17 were prepared with methanol at the final concentration of 1 mg/mL and then tested for antimicrobial activity against the thirty-three indicator strains. Besides, the remaining supernatant after the extraction of Anti-JFL21 or Anti-LQG17 were separately readjusted to pH 7, and the antimicrobial experiment was also carried out to verify whether the antimicrobial substances were sufficiently extracted. Antimicrobial activity was measured by implementing the agar disk diffusion assay mentioned above. Meanwhile, the antimicrobial spectrum of Anti-JFL21 and Anti-LQG17 against bacteria and fungi was compared to those of Nisin (1 mg/mL, Sigma) and polymyxin B (1 mg/mL, Sigma). The same amount of sterile water, methanol, and 0.02 mol/L HCl that applied to prepare samples were used as negative controls.

### Fourier Transformation Infra-Red (FTIR) Analysis

Fourier transformation infra-red spectroscopy analysis was performed to identify the structural groups of the purified biosurfactant using a Bruker Vertex 70v FTIR spectrometer (Bruker, Germany). In this experiment, 1 mg of Anti-JFL21 was ground with 100 mg of KBr in a pestle and pressed with load for 30 s to obtain translucent pellets. The FTIR spectra were collected at a frequency range from 4000 to 500 cm^–1^ (Sharma et al., 2015).

### Reversed-Phase HPLC Analysis

The powder of Anti-JFL21 and Anti-LQG17 was dissolved in an aqueous solution of 40% methanol at 1 mg/mL and filtered using a 0.22 μm pore filter (Millipore, United States). A 20 μL aliquot was injected into an Inertsil ODS-SP C18 column (4.6 mm ID × 25 cm L, 5 μm particle diameter) in the HPLC system (Shimadzu, Japan) to separate and identify the isoforms, according to published methods with some modifications (Yang et al., 2015). In brief, the equal volumes of 1 mg/mL standard surfactin, fengycin and iturin (purity of 90% or greater, Sigma-Aldrich, United States) were fully mixed and used to confirm the RP-HPLC fraction groups of three lipopeptide families. The mobile phases consisted of water (A) and acetonitrile (B), and all of them contained 0.1% trifluoroacetic acid (TFA). The detailed gradient strategy for the acetonitrile-water mobile phase system was as follows: 0–3 min, 45% acetonitrile to 50% acetonitrile; 3–8 min, 50% acetonitrile to 80% acetonitrile; 8–25 min, 80% acetonitrile to 100% acetonitrile; 25–30 min, 100% acetonitrile. The total flow rate of the mobile phases was kept at 0.8 mL/min, and the products were monitored by absorbance at 215 nm. All HPLC solvents were prepared fresh daily and filtered under vacuum before use.

### Matrix-Assisted Laser Desorption Ionization – Time of Flight Mass Spectrometry (MALDI-TOF MS) Analysis

Anti-JFL21 was further subjected to MALDI-TOF MS analysis and the methanol solvent was used as a negative control. The experiments were conducted using an ultrafleXtreme^TM^ MALDI-TOF MS instrument (Bruker Daltonics, Germany) equipped with a 337 nm pulsed nitrogen laser. All tested samples (2 μL) were mixed with an equal volume of the matrix (a saturated solution of α-cyano-4-hydroxycinnamic acid in 50% acetonitrile with 0.1% TFA), as described previously (Kim et al., 2010; Piewngam et al., 2018). The sample was spotted onto the gold-coated plate and air-dried. Then the target plate was loaded into an ultrafleXtreme^TM^ MALDI-TOF MS instrument (Bruker Daltonics, Germany). The mass spectrum was analyzed in the range of 500–3500 Da. MALDI-TOF MS/MS coupled with LIFT mode in the same spectrometer was used to analyze the fragment ions of the selected precursor ions for further characterization of the amino acid sequence. Other identification score criteria used were those recommended by the manufacturer.

### Isolation and Purification of Different Lipopeptides Family

To clarify which lipopeptide classes were responsible for antibacterial and antifungal activity, the family of iturin, fengycin, and surfactin were separated through Sephadex LH-20 gel filtration chromatography (GFC) (Kim et al., 2010; Zhang et al., 2013). *L. monocytogenes*, *A. hydrophila*, and *C. gloeosporioides* were used as indicator strains for quantifying antimicrobial effects since they were found to be the most sensitive indicator among Gram-positive pathogens, Gram-negative pathogens, and fungal pathogens, respectively. Briefly, the dried Anti-JFL21 was dissolved in 40% methanol (v/v) and prepared to a final concentration of 15 mg/mL, and filtered through a 0.2 μm membrane. To separate and purify the three lipopeptide family of Anti-JFL21, the filtrate (2 mL) of Anti-JFL21 was applied to a Sephadex LH-20 column (16 mm ID × 100 cm L) and fractionated by size-exclusion chromatography by using 40% methanol as eluent. A total of 100 fractions was collected (3 mL/tube) using a fraction collector (model DBS 100; Shanghai Hu Xi Analysis Instrument Factory Co. Ltd., Shanghai, China), and the flow rate of the eluent was kept at 0.5 mL/min. Then, each fraction was further detected using a UV spectrophotometer at 215 nm and analyzed by RP-HPLC, and tested for the antimicrobial activity.

### Determination of the Minimal Inhibitory Concentration of the Fengycins From Different Sources

After separation through GFC, the isolated active fractions containing only fengycin were pooled and dried with a vacuum freeze dryer. To further evaluate and compare the antimicrobial efficacy of fengycins isolated from Anti-JFL21 with the commercial fengycins standard (Sigma-Aldrich, United States), the minimal inhibitory concentration (MIC) was measured according to the agar disk diffusion assay (Sharma et al., 2015). *L. monocytogenes*, *A. hydrophila*, and *C. gloeosporioides* were used as indicator microorganisms, which were typically representative of Gram-positive pathogen, Gram-negative pathogen, and fungal pathogen, respectively. In brief, fengycins and its commercial standard was dissolved in methanol at a final concentration of 0.8 mg/mL and diluted by two-fold dilution to a variety of concentrations. Samples of different concentrations were then used for measuring of the antimicrobial activity against indicator strains and the MIC value was defined as the lowest concentration of samples that inhibits the visible growth of a tested strain after a chosen incubation period. The same amount of methanol was used as negative controls and the experiments were repeated independently three times.

### Biochemical Characterization of Fengycins

The powder of fengycins isolated from Anti-JFL21 was then dissolved in sterile phosphate-buffered saline (PBS) at pH 7.0 at a final concentration of 1 mg/mL and used for investigating the effect of temperature, pH, ultraviolet sterilization. The effect of temperature, pH, ultraviolet sterilization, enzymes, and chemical reagents on the antimicrobial activity of anti-JFL21 was evaluated by the agar disk diffusion assay using *L. monocytogenes* as an indicator strain (Sabaté and Audisio, 2013; Chalasani et al., 2015; Lee et al., 2016). The untreated sample was used as positive controls and its activity was defined as 100%. Negative controls were also performed without fengycins to subtract the inhibitory activity of the enzymes and chemical reagents.

In brief, the aliquots of fengycins were separately incubated under ultraviolet sterilization or in a water bath at different temperatures (37, 60, 80, and 100°C) for 2 h, and the residual antimicrobial activity was determined after cooling the treated samples to room temperature. Besides, the effect of pH on fengycins activity was examined over a pH range of 1.0–13.0 by adding 1N NaOH or HCl as appropriate. After incubation in the various pH for 2 h at 25°C, the samples were neutralized to pH 7.0 and its residual activity was measured. To evaluate stability to different enzymes, fengycins were incubated at 37°C for 2 h with 1 mg/mL (final concentration) of the following enzymes (Sigma, United States): cellulase, α-amylase, proteinase K, papain, bromelain, trypsin, and pepsin. The enzymes after incubation were inactivated by heating at 80°C for 10 min and then residual antimicrobial activities were measured. Also, the effects of various chemical reagents (EDTA, SDS, ZnSO_4_, and MnCl_2_) on fengycins activity were assayed after preincubating the fengycins with 1 mM (final concentration) of these reagents at 25°C and 100 r/min for 2 h.

### Statistical Analysis

The diameters of the zones of inhibition against various indicator strains were expressed as mean values ± SD. All the assays were repeated at least in three separate experiments. The student’s *t*-test was utilized to determine the statistically significant difference in the residual anti-*Listeria* activity after the various treatments. The *P*-values of less than 0.05 (*p* < 0.05) were considered significant.

## Results

### Measurement of Drug Resistance of the Probiotics and Pathogens

To investigate the drug resistance of probiotics and pathogens used in this study, the inhibitory effects of the twelve commonly used antibiotics against thirty-three indicator strains were examined. The negative controls (sterile water, anhydrous ethanol, DMSO, and 0.01 mol/L HCl) did not show any antimicrobial ability against all the indicator strains (data not shown), which suggest that the solvents itself did not affect the antimicrobial activity of the antibiotics against the tested indicator strains. As presented in Table 1, all the pathogenic microorganisms used in this study were resistant to several different antibiotics, indicating all of them were multidrug-resistant. In particular, all pathogens except for *C. sakazakii* and *K. pneumoniae* were resistant to at least six antibiotics. Furthermore, conventional antibiotics usually did not have selective antimicrobial effects, and they inhibit the growth of probiotics as well as pathogens. Thus, it is important to develop alternative antibiotics that are less toxic to most probiotics but can inhibit or kill pathogens extensively and potently.

### Characterization of *Bacillus* Strains Isolated From Fermented Food and Seafood Products

**FIGURE 1:**
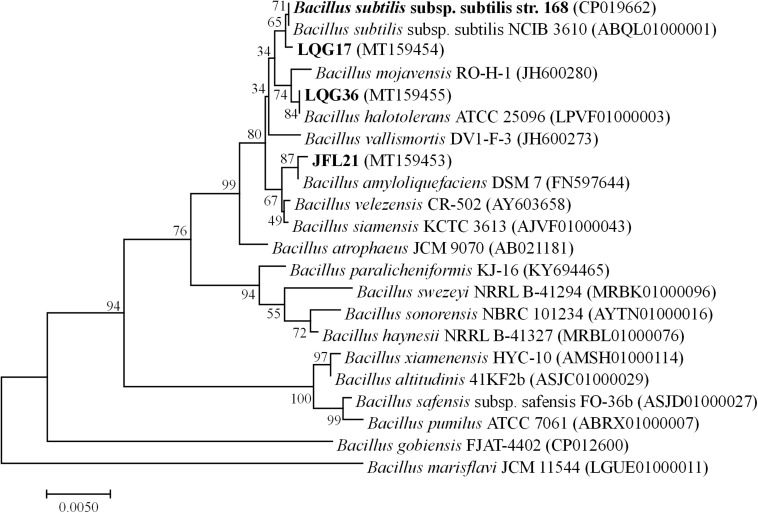
Phylogenetic tree based on 16SrDNA gene sequences from four *Bacillus* strains using the Neighbor-joining method (1000 bootstrap replicates).

A phylogenetic tree based on the 16S rDNA sequences of the four *Bacillus* spp. was constructed and shown in Figure 1. As can be seen, the strain *Bacillus* sp. JFL21 was assigned in a node with 99.86% of support with *B. amyloliquefaciens* DSM 7, while the strain *Bacillus* sp. LGQ17 was clustered together with the members of other *B. subtilis* groups supported by a sequence similarity of 99.93%. Moreover, *Bacillus* sp. LGQ36 was designated as *B. halotolerans* ATCC 25096 because their 16S rDNA gene sequences were 100% identical.

### Comparison of the Antimicrobial Activities of Metabolites From Different *Bacillus* spp.

**FIGURE 2:**
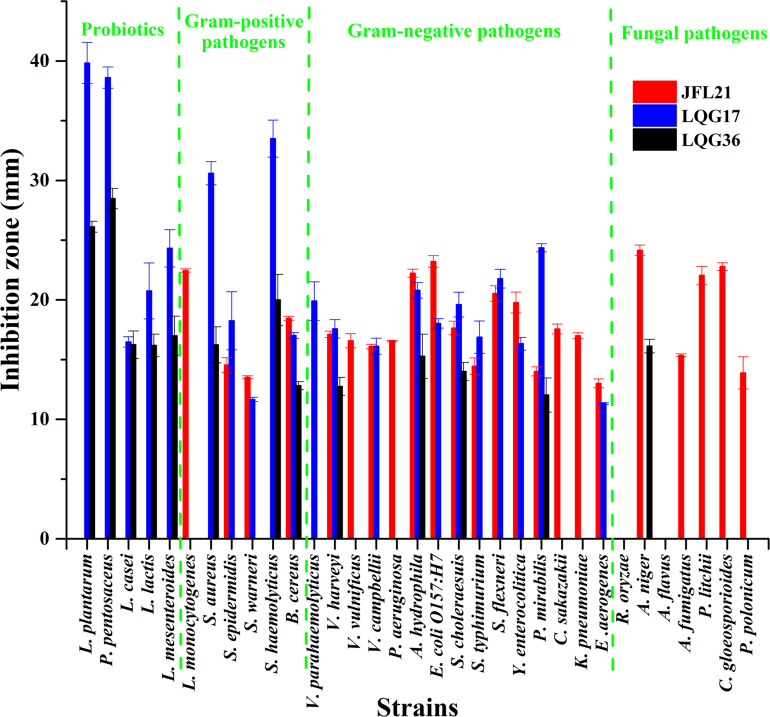
Antimicrobial spectrum of cell-free supernatants from different *Bacillus* strains. JFL21, LQG17, and LQG36 were referred to as the cell-free supernatants produced by *Bacillus amyloliquefaciens* JFL21, *Bacillus natto* LQG17, and *Bacillus halotolerans* LQG36, respectively.

**FIGURE 3:**
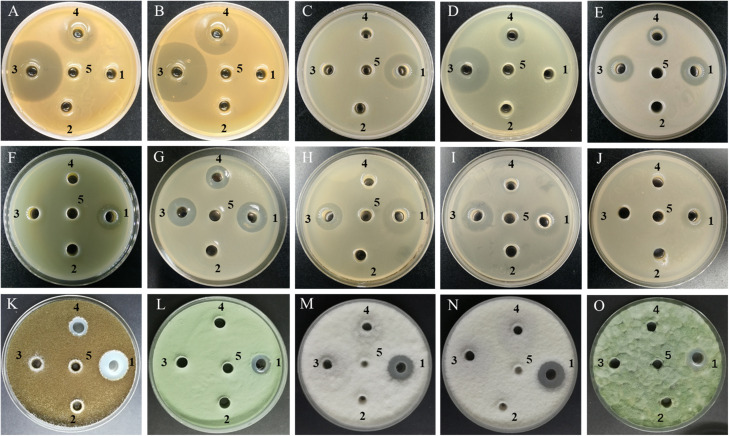
Antimicrobial activity of cell-free supernatants from different *Bacillus* strains against some representative indicator microorganisms. **(A)** *L. plantarum*; **(B)** *L. pentosus*; **(C)** *L. monocytogenes*; **(D)** *S. aureus*; **(E)** *B. cereus*; **(F)** *P. aeruginosa*; **(G)** *A. hydrophila*; **(H)** *E. coli* O157:H7; **(I)** *S. typhimurium*; **(J)** *C. sakazakii*; **(K)** *A. niger*; **(L)** *A. fumigatus*; **(M)** *P. litchii*; **(N)** *C. gloeosporioides*; **(O)** *P. polonicum*; 1–4, the cell-free supernatants produced by *Bacillus amyloliquefaciens* JFL21, *Bacillus subtilis* 168, *Bacillus subtilis* LQG17, and *Bacillus halotolerans* LQG36, respectively; 5, the uninoculated Landy medium.

As presented in Figures 2, 3, the CFS produced by *B. subtilis* LGQ17 and *B. halotolerans* LGQ36 had an obvious inhibitory effect on the majority of indicators except for pathogenic fungi, while the CFS produced by *B. amyloliquefaciens* JFL21 exhibited a marked antimicrobial activity toward the majority of indicators except for probiotics. Among them, the CFS produced by *B. amyloliquefaciens* JFL21 exhibited broader inhibitory spectrum toward pathogens but showed no inhibitory effect on probiotics, and thus has higher application value. However, the CFS produced by *B. subtilis* 168 failed to show any antibacterial or antifungal effect against all indicators, which may be due to its poor capability to produce antimicrobial substances. Based on the antimicrobial spectrum and inhibitory efficacy, the antimicrobial substance produced by *B. amyloliquefaciens* JFL21 and *B. subtilis* LGQ17 were selected for the additional extraction and characterization.

### Inhibitory Spectrum of the Bioactive Substance Produced From *B. amyloliquefaciens* JFL21 and *B. subtilis* LGQ17

As showed in Table 2, Anti-JFL21 exhibited significant antibacterial and antifungal activity against most indicators while Anti-LQG17 showed a weak antibacterial effect against a few indicators. To further check whether the antimicrobial substances were adequately extracted, the remaining supernatant after the extraction of Anti-JFL21 or Anti-LQG17 were also tested for the antimicrobial activity. The result revealed that the remaining supernatant after the extraction of Anti-LQG17 (CFS-LQG17) still has an obvious antibacterial effect on many indicators, which indicated Anti-LQG17 maybe not the primary antimicrobial substances produced by *B. subtilis* LGQ17 and the main active ingredients need to be clarified by subsequent experiments. However, the remaining supernatant after the extraction of Anti-JFL21 (CFS- JFL21) did not inhibit the growth of every indicator (data not shown), confirming the antimicrobial substances were sufficiently extracted from the CFS of *B. amyloliquefaciens* JFL21. Additionally, the antimicrobial activity of Anti-JFL21 was also compared with those of commercially available nisin and polymyxin B, which were represented as bacteriocin and lipopeptide, respectively (Table 2). As can be observed, Anti-JFL21 not only exhibited unique advantages in inhibiting the growth of a broad range of fungal pathogens but also showed a wider and stronger antibacterial activity toward Gram-positive pathogens when compared with polymyxin B and Nisin. What’s more, although Anti-JFL21 also inhibited the growth of some probiotics, the inhibitory activity against probiotics was significantly lower when compared to pathogens. These results suggest that Anti-JFL21 could be expected to a promising alternative source of biological preservatives in the food industry for controlling various multidrug-resistant foodborne pathogens and extending the shelf-life of food products.

**TABLE 2:** Inhibition spectrum of Anti-JFL21 and Anti-LQG17^*a*^.

Indicator strains	Inhibition zone (mm)				
	Anti-JFL21 (1 mg/mL)	Anti-LQG17 (1 mg/mL)	CFS-LQG17 (100 ul)^*b*^	Polymyxin B (1 mg/mL)	Nisin (1 mg/mL)
**Probiotics**					
*L. plantarum*	14.36 ± 1.22	12.46 ± 0.89	29.24 ± 0.87	−	15.07 ± 0.35
*P. pentosaceus*	−	−	26.12 ± 0.56	−	14.13 ± 0.62
*L. casei*	13.22 ± 0.67	−	−	−	21.76 ± 0.96
*L. lactis*	−	−	15.33 ± 2.25	−	17.34 ± 0.74
*L. mesenteroides*	−	−	20.59 ± 1.37	−	19.51 ± 1.69
**G^+^ pathogen**					
*L. monocytogenes*	22.96 ± 0.74	−	−	−	−
*S. aureus*	14.51 ± 1.48	−	25.22 ± 1.14	−	−
*S. epidermidis*	13.22 ± 0.46	−	14.12 ± 0.57	12.17 ± 0.31	17.15 ± 0.54
*S. warneri*	12.98 ± 0.51	−	13.02 ± 0.79	14.22 ± 1.04	−
*S. haemolyticus*	−	−	18.02 ± 0.66	−	−
*B. cereus*	19.80 ± 0.25	12.99 ± 0.49	14.88 ± 0.42	−	−
**G^–^ pathogen**					
*V. parahaemolyticus*	−	−	21.12 ± 0.85	15.34 ± 1.63	−
*V. harveyi*	15.65 ± 1.20	−	−	22.45 ± 0.19	−
*V. vulnificus*	−	−	−	−	−
*V. campbellii*	14.55 ± 1.67	−	−	16.42 ± 0.47	−
*P. aeruginosa*	20.25 ± 0.57	−	−	−	−
*A. hydrophila*	22.72 ± 0.34	11.98 ± 0.73	20.96 ± 0.08	21.13 ± 0.87	−
*E. coli* O157:H7	21.45 ± 0.96	−	13.08 ± 1.45	15.36 ± 0.95	−
*S. choleraesuis*	19.65 ± 0.42	−	12.66 ± 0.75	15.74 ± 0.32	−
*S. typhimurium*	17.94 ± 1.03	−	−	15.89 ± 0.45	−
*S. flexneri*	15.73 ± 2.86	−	20.02 ± 0.17	15.93 ± 0.22	−
*Y. enterocolitica*	16.85 ± 2.51	−	−	17.96 ± 2.01	−
*P. mirabilis*	20.71 ± 0.11	10.02 ± 1.15	17.46 ± 0.32	−	−
*C. sakazakii*	20.57 ± 0.92	−	−	14.86 ± 1.15	−
*K. pneumoniae*	17.34 ± 0.34	−	−	17.03 ± 0.84	−
*E. aerogenes*	16.58 ± 1.54	−	−	14.65 ± 0.73	−
**Pathogenic fungi**					
*R. oryzae*	−	−	−	−	−
*A. niger*	21.76 ± 0.15	−	−	−	−
*A. flavus*	18.28 ± 0.75	−	−	−	−
*A. fumigatus*	20.13 ± 0.68	−	−	−	−
*P. litchii*	16.55 ± 0.91	−	−	−	−
*C. gloeosporioides*	22.24 ± 0.49	−	−	−	−
*P. polonicum*	19.21 ± 1.13	−	−	−	−

*^*a*^−: no inhibition zone; ^*b*^CFS-LQG17: the remaining supernatant after the extraction of Anti-LQG17.*

### Structure Analysis of Crude Lipopeptides by FTIR

**FIGURE 4:**
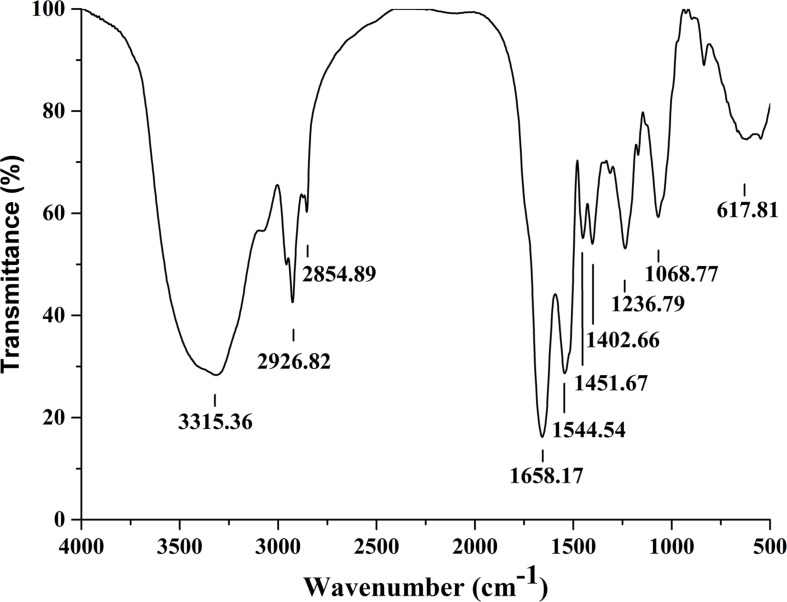
Fourier transformation infra-red spectra analysis of the lipopeptides mixture Anti-JFL21 obtained from *Bacillus amyloliquefaciens* JFL21.

Fourier transformation infra-red spectral peaks of Anti-JFL21 were exhibited in Figure 4. The broader absorption peak of 3315.36 cm^–1^ indicates the presence of –OH or –NH groups. Peaks at 2926.82 cm^–1^, 2854.89 cm^–1^, 1451.67 cm^–1^, and 1402.66 cm^–1^ confirm the –C–H stretching (−CH3, −CH2) of the aliphatic chain of the lipid. A similar stretching for –C–H of lipid was also found by Pradhan et al. (2013) and Al-Wahaibi et al. (2014). The presence of amide bond (617.81 cm^–1^), N–H bending of secondary amides (1544.54 cm^–1^), and the carbonyl group (C = O) of amide (1658.17 cm^–1^) confirms the peptide fraction in the sample. C–O bending of esters was characterized by the peaks at 1236.79 cm^–1^ and 1068.77 cm^–1^. The same bending and stretching were reported for peptide groups in the previous research (Pradhan et al., 2013; Sharma et al., 2015; Varjani and Upasani, 2016). Thus, the nature of the antimicrobial substance produced by *B. amyloliquefaciens* JFL21 was speculated as cyclic lipopeptide compounds.

### Lipopeptides Quantification by RP-HPLC Analysis

**FIGURE 5:**
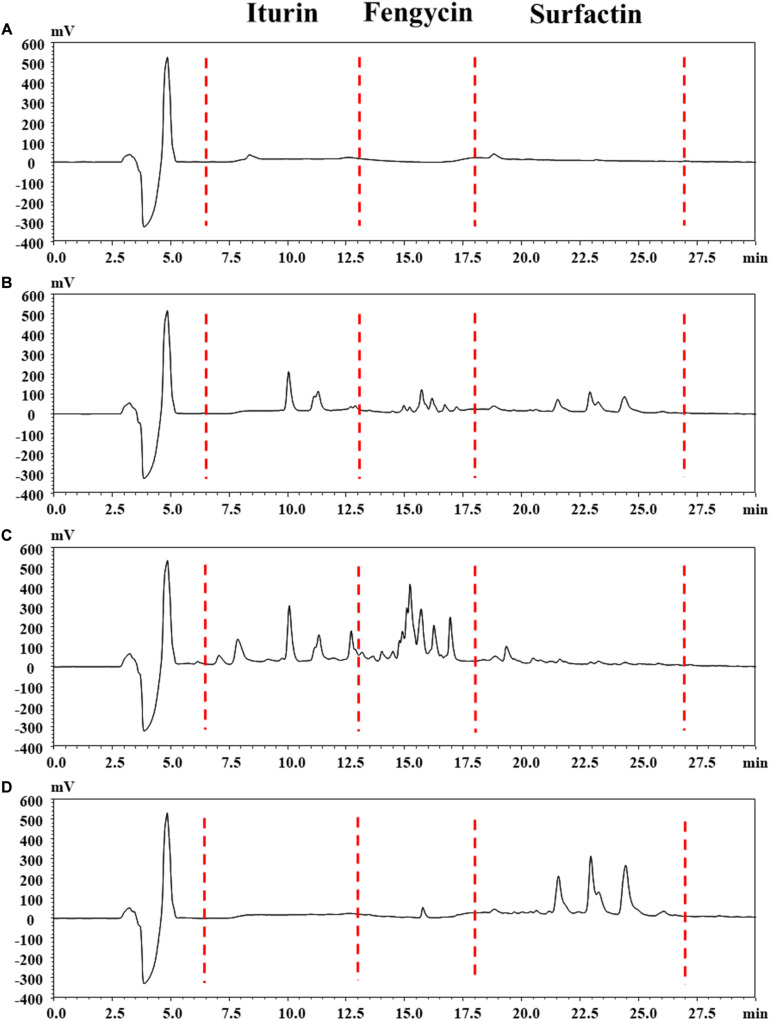
Reversed phase HPLC chromatogram analysis of the lipopeptides mixture Anti-JFL21 and Anti-LQG17. **(A)** methyl alcohol; **(B)** complexes with final concentrations of 0.33 mg/mL of commercial standard iturin, fenycin, and surfactin; **(C)** 1 mg/mL Anti-JFL21 obtained from *Bacillus amyloliquefaciens* JFL21; **(D)** 1 mg/mL Anti-LQG17 obtained from *Bacillus natto* LQG17.

The reversed-phase HPLC (RP-HPLC) analysis was carried out to characterize the structural properties of Anti-JFL21 and Anti-LGQ17, and the results were presented in Figure 5. The peaks at retention time 4.86, 8.37, and 18.81 min maybe methanol solvent or impurity peak (Figure 5A). The retention times of iturin, fengycin, and surfactin were identified in the ranges of 6.5–13, 13–18, and 18–27 min, respectively, according to our previous results (Figure 5B). For the lipopeptides extract Anti-JFL21, three peaks clusters were observed on the RP-HPLC profile which exhibited similar retention times as the three standard groups (Figure 5C). The RP-HPLC profiles of Anti-JFL21 showed the major peaks which corresponded to iturin and fengycin families, while traces of surfactin families were also detected but the content was relatively low. Furthermore, the peaks of the iturin and fengycin families in Anti-JFL21 were relatively more than those of the three standards, which may be due to the more analogs or homologs of three lipopeptide families in Anti-JFL21. Meanwhile, RP-HPLC analysis also revealed that Anti-LGQ17 was mainly composed of four surfactin analogs (Figure 5D). These results suggested that different *Bacillus* species could synthesize different CLPs family, and the antimicrobial properties of these CLPs exhibited significant differences.

### Identification of the Active Compounds by MALDI-TOF MS

**FIGURE 6:**
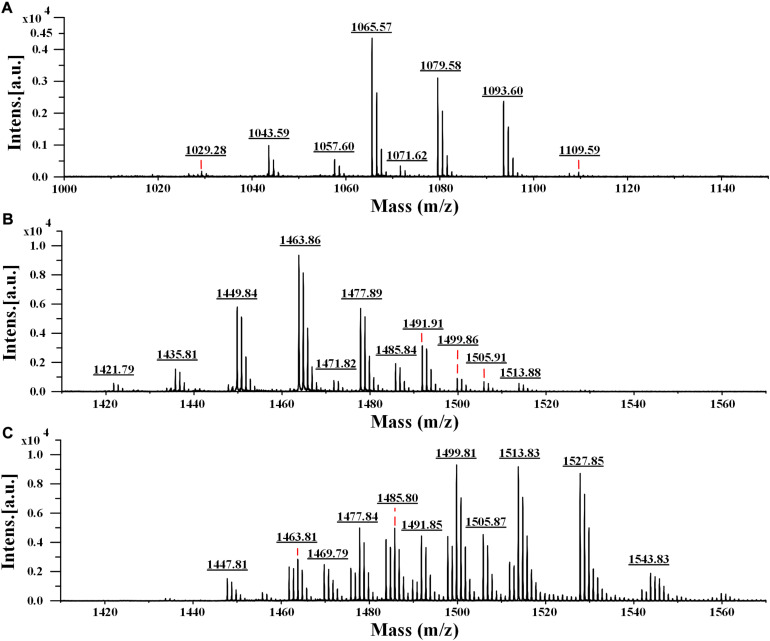
MALDI-TOF MS analysis of the lipopeptides mixture Anti-JFL21 and the commercial fengycin standard. **(A)** the peaks of Anti-JFL21 in the range of *m/z* = 1000–1150 Da; **(B)** the peaks of Anti-JFL21 in the range of *m/z* = 1410–1570 Da; **(C)** the peaks of the commercial fengycin standard in the range of *m/z* = 1410–1570 Da.

To determine the accurate molecular mass, Anti-JFL21 was further subjected to MALDI-TOF MS analysis. Anti-LGQ17 was not selected for further analysis since it did not show significant antimicrobial activity. As shown in Figure 5, the MALDI-TOF MS analysis result of Anti-JFL21 showed two well-resolved groups of peaks at *m/z* values range of 1029.28–1109.59 Da (Figure 6A) and 1421.79–1513.88 Da (Figure 6B), which could be, respectively, attributed to the isomers of iturin and fengycin, as previously described (Torres et al., 2015; Yang et al., 2015; Sarwar et al., 2018). As can be observed in Figure 6A, the protonated molecular ion ([M + H]^+^) peaks of the iturin A homologs were detected at *m/z* 1029.28, 1043.59, 1057.60, and 1071.62 Da, and the peaks with the differences of 14 Da may correspond to the molecular weight of one CH_2_ group. Besides, peaks in the *m/z* of 1065.57, 1079.58, and 1093.60 Da may be assigned as the sodium adducts ([M + Na]^+^) of C_14_, C_15_, and C_16_ homologs of iturin A, and *m/z* = 1109.59 Da could be attributed to the potassium adducts ([M + K]^+^) for C_16_ iturin. However, the absence of a typical peak of surfactin (e.g., *m/z* 1008, 1022, 1034, 1046, and 1058) may be due to its low relative content in the sample, which is consistent with the results of RP-HPLC analysis. On the other hand, several fengycin homologs were detected as [M + H]^+^ at *m/z* 1421.79, 1435.81, 1449.84, 1463.86, 1477.89, and 1491.91 Da (Figure 6B), which may correspond to the different fatty acid length. Moreover, the [M + Na]^+^ and [M + K]^+^ adducts of fengycin isoforms were also observed at *m/z* 1471.82, 1485.84, 1499.86, and 1513.88 Da (Figure 6B). Compared with the groups of fengycin peaks of Anti-JFL21, the commercial fengycin standard was also identified with many of the same ions of [M + H]^+^, [M + Na]^+^, and [M + K]^+^, such as the 1463.81, 1477.84, 1485.80, 1491.85, 1499.81, and 1505.87 Da (Figure 6C). In addition, the difference between the ion of 1447.81 Da detected in the commercial fengycin standard and the 1449.84 Da identified in Anti-JFL21 only 2 Da, which may be due to the presence of a double bond in fatty acids chain (Pathak et al., 2012; Sa et al., 2018). The major *m/z* peaks of Anti-JFL21 and the fengycin standard were summarized in the Table 3.

**TABLE 3:** Possible assignments of major *m/z* peaks of Anti-JFL21 and the commercial fengycin standard^*a*^.

Samples	Detected precursor ions (*m/z*)			Two fingerprint ions (*m/z*)	Possible assignment^*b*^	Amino acid at position 6 and 10
	[M + H]^+^	[M + Na]^+^	[M + K]^+^			
Anti-JFL21	1029.28	−	−	/	C_13_ iturin A	/
	1043.59	1065.57	−	/	C_14_ iturin A	/
	1057.60	1079.58	−	/	C_15_ iturin A	/
	1071.62	1093.60	1109.59	/	C_16_ iturin A	/
	1421.79	−	−	952.25, 1066.42	C_14_ fengycin A2	Ala^6^, Val^10^
	1435.81	−	−	952.35, 1066.44 966.37, 1080.48	C_15_ fengycin A2 C_14_ fengycin A1	Ala^6^, Val^10^ Ala^6^, Ile^10^
	1449.84	1471.82	−	952.30, 1066.41 966.31, 1080.40 980.34, 1094.45	C_16_ fengycin A2 C_15_ fengycin A1 C_14_ fengycin B2	Ala^6^, Val^10^ Ala^6^, Ile^10^ Val^6^, Val^10^
	1463.86	1485.84	−	952.32, 1066.44 966.35, 1080.46	C_17_ fengycin A2 C_16_ fengycin A1	Ala^6^, Val^10^ Ala^6^, Ile^10^
	1477.89	1499.86	−	952.43, 1066.50 966.37, 1080.48 980.39, 1094.51 994.39, 1108.52	C_18_ fengycin A2 C_17_ fengycin A1 C_16_ fengycin B2 C_15_ fengycin B1	Ala^6^, Val^10^ Ala^6^, Ile^10^ Val^6^, Val^10^ Val^6^, Ile^10^
	1491.91	1513.88	−	952.26, 1066.18 966.19, 1080.16 980.20, 1094.20 994.35, 1108.50	C_19_ fengycin A2 C_18_ fengycin A1 C_17_ fengycin B2 C_16_ fengycin B1	Ala^6^, Val^10^ Ala^6^, Ile^10^ Val^6^, Val^10^ Val^6^, Ile^10^
	1505.91	−	−	980.39, 1094.47 994.42, 1108.53	C_18_ fengycin B2 C_17_ fengycin B1	Val^6^, Val^10^ Val^6^, Ile^10^
fengycin standard	1447.81	1469.79	−	966.45, 1080.55 994.47, 1108.55	*C_15_ fengycin A1 *C_13_ fengycin B1	Ala^6^, Ile^10^ Val^6^, Ile^10^
	1463.81	1485.80	−	966.37, 1080.46 994.38, 1108.48	C_16_ fengycin A1 C_14_ fengycin B1	Ala^6^, Ile^10^ Val^6^, Ile^10^
	1477.84	1499.81	−	966.39, 1080.47 994.40, 1108.49	C_17_ fengycin A1 C_15_ fengycin B1	Ala^6^, Ile^10^ Val^6^, Ile^10^
	1491.85	1513.83	−	966.34, 1080.45 994.33, 1108.44	C_18_ fengycin A1 C_16_ fengycin B1	Ala^6^, Ile^10^ Val^6^, Ile^10^
	1505.87	1527.85	1543.83	994.34, 1108.46	C_17_ fengycin B1	Val^6^, Ile^10^

^*a*^−, no detected; /, not tested or studied; *, indicates one double bond in the fatty acid chain. ^*b*^Possible assignments based on Pathak et al. (2012), Torres et al. (2015); Yang et al. (2015), and Sarwar et al. (2018).

### Characterization of Fengycin Isoforms by MALDI-TOF MS/MS

**FIGURE 7:**
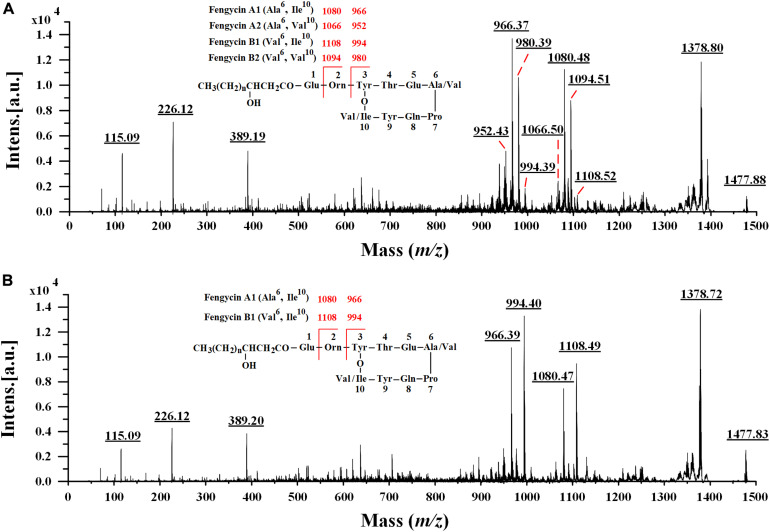
Comparison of MALDI-TOF MS/MS spectra of the lipopeptides mixture Anti-JFL21 and the commercial fengycin standard. MS/MS spectra of the precursor ion of 1477.88 Da from Anti-JFL21 **(A)** compared to the precursor ion of 1477.84 Da from the commercial fengycin standard **(B)**.

In the earlier literature, fengycins have been mainly divided into fengycin A and fengycin B, which are characterized by the existence of Ala and Val residues at position 6 of the peptide ring, respectively (Vanittanakom et al., 1986; Wang et al., 2004). Recently, a new variant of fengycin, termed as fengycin A2 or fengycin B2 with the presence of Val instead of Ile at position 10 was also observed (Pathak et al., 2012; Cochrane and Vederas, 2016). Since many fengycin isoforms may have the same nominal mass and molecular formula, it is difficult for MALDI-TOF MS to definitely discriminate different fengycin isomers. To obtain more precise amino acid sequence information, the fengycin protonated parent ions ([M + H]^+^) of Anti-JFL21 together with the commercial fengycin standard were selected for further MALDI-TOF MS/MS analysis. The representative MS/MS spectra of the precursor ion of 1477 Da from Anti-JFL21 compared to the fengycin standard was displayed in Figure 7.

In the MS/MS spectrum of the precursor ion of 1477.88 Da (Figure 7A), it can be quickly identified as C_18_ fengycin A2 (Ala^6^, Val^10^), C_17_ fengycin A1 (Ala^6^, Ile^10^), C_15_ fengycin B1 (Val^6^, Ile^10^) and C_16_ fengycin B2 (Val^6^, Val^10^) based on the presence of four typical paired product ions of 952 and 1066 Da, 966 and 1080 Da, 980 and 1094 Da, and 994 and 1108 Da, respectively (Wang et al., 2004; Pathak et al., 2012). Similarly, two paired product ions of 966 and 1080 Da, and 994 and 1108 Da were appeared in MS/MS spectrum of the parent ion at *m/z* 1477.84 Da (Figure 7B), which enable it to be assigned corresponding to C_17_ fengycin A1 and C_15_ fengycin B1. Unlike Anti-JFL21, the fengycin standard were failed to detect the two paired specific fingerprint ions of 952 and 1066 Da, and 980 and 1094 Da (Figure 7B and Table 3), which indicated the fengycin standard was only composed of fengycin A1 and fengycin B1. Besides, the product ion of 1378 Da may be resulted from the loss of Val while a series of fragment ions at *m/z* 389 Da, 226 Da, and 115 Da may specifically represented the sequence of Pro-Gln-Tyr, Pro-Gln, and Pro-H_2_O, respectively (Yu et al., 2012; Yang et al., 2015). In addition, other fengycin precursor peaks, fingerprint ions, and structural assignments were summarized and showed in Table 3.

### Isolation and Purification of Lipopeptides

**FIGURE 8:**
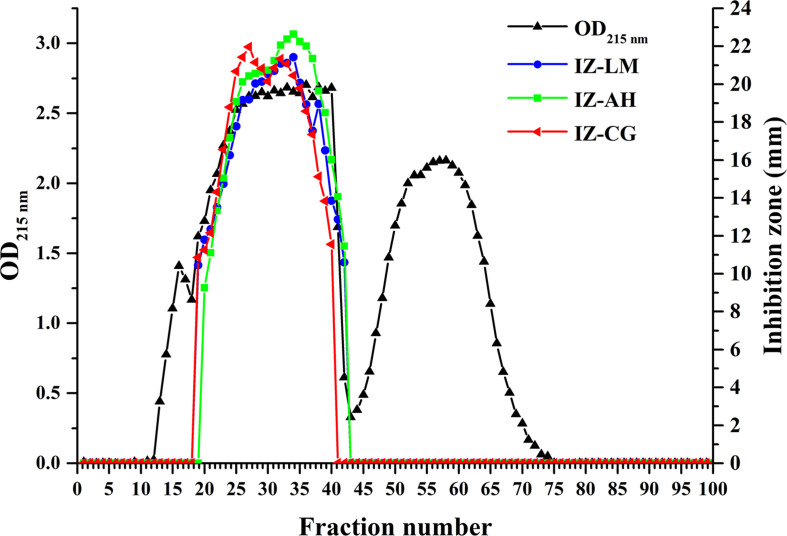
Gel filtration chromatography and antimicrobial activity of the different fractions separated from the lipopeptides mixture Anti-JFL21 produced by *Bacillus amyloliquefaciens* JFL21. IZ-LM, IZ-AH, and IZ-CG were referred to as the inhibition zone of the different fractions separated from Anti-JFL21 against *L. monocytogenes*, *A. hydrophila*, and *C. gloeosporioides*, respectively.

To assess the antimicrobial contribution of individual lipopeptide classes, Anti-JFL21 was subjected to further isolation and purification by GFC. As shown in Figure 8, the spectrophotometer analysis at 215 nm indicated the possible presence of lipopeptides in the fractions from 13 to 66, which was also confirmed by RP-HPLC (data not shown). Besides, RP-HPLC results revealed that the fractions in the range of 13–18, 19–42, and 43–72 were mainly identified as single surfactin, fengycin, and iturin families, respectively (Supplementary Figure 1). The fractions containing fengycins exhibited the primary antimicrobial activity against *L. monocytogenes*, *A. hydrophila*, and *C. gloeosporioides*, whereas the rest of the fractions comprising only surfactins or iturins did not present any antagonistic effect on the three indicator strains (Figure 8 and Supplementary Figure 2). These results demonstrated that fengycins play a key role in antimicrobial activity.

### Comparison of Minimal Inhibitory Concentration of the Fengycins Isolated From Anti-JLF21 and the Commercial Fengycins Standard

To compare the antimicrobial efficacy of the fengycins isolated from Anti-JFL21 with the commercial fengycins standard, their MIC was determined and compared using *L. monocytogenes*, *A. hydrophila*, and *C. gloeosporioides* as the indicator strains (Table 4). The result showed that the fengycins isolated from Anti-JFL21 could display the antimicrobial effect toward *L. monocytogenes*, *A. hydrophila*, and *C. gloeosporioides* at a low MIC range of 25–50 ug/mL. Besides, the commercial fengycins standard was found to active against *C. gloeosporioides* at low MIC concentration (25 ug/mL) but fail to inhibit the growth of *L. monocytogenes* and *A. hydrophila* even at the highest tested concentration (800 μg/ml). These results suggest that the fengycins isolated from Anti-JFL21 are more efficient in inhibiting pathogenic bacteria as compared to the commercial fengycins standard, which may be contributed to the greater variety of structural analogs, especially in the variation at position 10 (Val/Ile) (Table 3).

**TABLE 4:** Minimum inhibitory concentration of different fengycin sources against some representative indicator strains.

Indicator strains	MIC (ug/ml)	
	Fengycins isolated from Anti-JFL21	The commercial Fengycins standard
*L. monocytogenes*	25	>800
*A. hydrophila*	50	>800
*C. gloeosporioides*	25	25

### Biochemical Characterization of Fengycins

To evaluate the possible application in the food industry, the antimicrobial stability of fengycins produced by *B. amyloliquefaciens* JFL21 under various conditions was determined. *L. monocytogenes*, an important foodborne pathogen was used as an indicator strain. As shown in Table 5, the fengycins did not show loss of its inhibitory activity against *L. monocytogenes* after the treatment for 2 h under the ultraviolet sterilization, over a pH range of 5.0 to 9.0, or at temperatures below 80°C. Although there was a very significant reduction in the activity after incubation for 2 h at 100°C, pH 1.0, and pH 13.0, the residual activity remained 90, 85, and 72%, respectively. Thus, our results indicate that the fengycins were resistant to the various harsh conditions and the sterilization process such as commercial pasteurization and ultraviolet sterilization, and thus can be used as a natural preservative in the food industry.

**TABLE 5:** Effect of enzymes, chemical reagents, and physical factors on fengycins anti-*Listeria* activity^*a*^.

Factors	Treatment for 2 h	Relative activity (%)^*a*^
Control	Untreated	100
pH	1	85 ± 3.8**
	3	93 ± 2.6*
	5	99 ± 1.1
	7	102 ± 1.6
	9	101 ± 0.7
	11	88 ± 3.2**
	13	72 ± 5.9**
Heat	37°C	99 ± 1.9
	60°C	100 ± 0.5
	80°C	97 ± 1.5
	100°C	90 ± 4.3*
Light	Ultraviolet	101 ± 0.3
Enzymes (1 mg/mL)	Cellulase	104 ± 2.6
	α-Amylase	99 ± 1.4
	Proteinase K	98 ± 0.5
	Papain	100 ± 2.7
	Bromelain	101 ± 1.0
	Trypsin	100 ± 0.2
	Pepsin	98 ± 5.3
Chemical reagents (1 mM)	EDTA	102 ± 2.4
	SDS	97 ± 1.8
	MnCl_2_	100 ± 1.1
	ZnSO_4_	92 ± 6.4

^*a*^The relative antimicrobial activity was measured by using *L. monocytogenes* as an indicator strain. Values are given as mean ± SD from triplicate determinations (*n* = 3). The value represents the mean ± SE of triple determinations. *p < 0.05, **p < 0.01.

## Discussion

The increased emergence and evolution of multiple drug-resistant foodborne pathogens in recent decades has become a worldwide vexing problem of public concern. In this work, antibiotics susceptibility test results indicated that all foodborne pathogens used in the current study were multidrug-resistant according to the recent drug-resistance definition (Table 1; Magiorakos et al., 2012). Likewise, Lee et al. (2017) disclosed that 27 (82%) of 33 *L. monocytogenes* isolates collected from ready-to-eat seafood and food processing environments were resistant to at least four antibiotics. Besides, a high proportion of multidrug-resistant isolates from food was also commonly reported in other foodborne pathogens such as *Y. enterocolitica* (66/70, 94.3%) (Ye et al., 2015), *Salmonella* (30/42, 71.4%) (Tirziu et al., 2015), and *S. aureus* (62/93, 66.67%) (Ma et al., 2018). Thus, the development of new antimicrobial agents or other alternative strategies is required promptly.

*Bacillus* is an important genus producing a wide array of bioactive secondary metabolites, including lipopeptides, polyketides, lantibiotics, siderophores, lytic enzymes, and peptides (Cawoy et al., 2015; Dimkić et al., 2017). Moreover, the majority of *Bacillus* spp. such as *B. subtilis*, *B. amyloliquefaciens*, and *B. licheniformis* have been generally recognized as safe microorganisms for application in the food industry and considered to be important biological control agents (Torres et al., 2015; Lastochkina et al., 2019). In this study, three *Bacillus* isolates from various food sources, namely *B. amyloliquefaciens* JFL21, *B. subtilis* LQG17, and *B. halotolerans* LQG36 were obtained and their antimicrobial properties were compared. Results revealed that the CFS produced by these *Bacillus* strains exhibited different inhibitory spectrum or inhibitory efficacy against the thirty-three indicators (Figures 2, 3), which suggested that different *Bacillus* species or isolates may be capable of producing different kinds or amounts of bioactive metabolites. Hence, screening and characterization of the *Bacillus* spp. with remarkable biological properties still receives a great scientific interest due to the application potential of these strains and their active metabolites (Li et al., 2015).

In recent years, CLPs have received considerable attention because of their efficient biosurfactant and antimicrobial properties (Perez et al., 2017; Sarwar et al., 2018). In this work, the lipopeptides mixture Anti-JFL21 not only exhibited strong antimicrobial activities against the majority of multidrug-resistant foodborne pathogens but also showed relatively low toxicity to most probiotics (Figures 2, 3). This may be since all probiotics used in this study belongs to lactic acid bacteria (LAB), and Anti-JFL21 have a relatively weak inhibitory effect on LAB. The similar phenomenon was also observed by Lee et al. (2016), who reported that the lipopeptides mixture containing four surfactin and four bacillomycin D analogs, inhibited the growth of many Gram-positive pathogens (e.g., *B. cereus* and *L. monocytogenes*) and fungal pathogen (e.g., *A. nidulans* and *F. moniliforme*), but it did not inhibit LAB such as *L. plantarum* and *L. lactis*. Moreover, Todorov et al. (2018) also found that *L. plantarum* has a natural high resistance to a glycopeptide antibiotic when compared to *L. monocytogenes*, which was attributed to the presence of D-Ala-D-Lactate in its peptidoglycan rather than in the D-Ala-D-Ala dipeptide. Thus, it is speculated that the weak inhibitory effect of lipopeptides Anti-JFL21 on LAB may be due to the difference of cell wall composition between LAB and other indicator bacteria. However, the specific mechanism of the relatively weak bacteriostatic effect of CLPs on LAB still needs to be further explored.

Historically, the elucidation of specific effective antimicrobial components is crucial to the accuracy of the drug application or antiseptic addition. In this research, the family of surfactin, fengycin, and iturin were successfully separated by GFC, and the results of the antimicrobial test demonstrated that fengycins might play a major role in the antibacterial and antifungal activity of Anti-JFL21 (Figure 8 and Supplementary Figures 1,2). To our knowledge, this study is the first report that fengycin is a primary active compound of *B. amyloliquefaciens* against a broad range of foodborne pathogens including Gram-positive pathogens, Gram-negative pathogens, and fungal pathogens. However, unlike some previous studies, which displayed that surfactin and iturin presented important antibacterial and antifungal activity, respectively (Loiseau et al., 2015; Narendra Kumar et al., 2017; Sudarmono et al., 2019). Surfactins produced by *B. subtilis* LQG17 showed a weak antibacterial effect against few bacterial indicators, and surfactins and iturins produced by *B. amyloliquefaciens* JFL21 were not able to inhibit either bacteria or fungi. This may be due to the production and structural diversity of each lipopeptide family are influenced by the various *Bacillus* species or isolates (Sabaté and Audisio, 2013; Fan et al., 2017; Sudarmono et al., 2019), and the effective isoforms of surfactin and iturin synthesized by *B. subtilis* LQG17 or *B. amyloliquefaciens* JFL21 was few or not enough to reach the antimicrobial concentration. This phenomenon was also found by Sabaté and Audisio (2013), who reported that the surfactin produced by *B. subtilis* C4 inhibited the *L. monocytogenes* with a concentration of 0.125 mg/mL while 1 mg/mL of the surfactin produced by *B. subtilis* M1 was necessary to inhibit the pathogen.

Indeed, it is well known that different isoforms and homologs of the lipopeptide family could exhibit different properties and activities, which depend in particular on the chain length (Ben Ayed et al., 2014). In this work, RP-HPLC and MALDI-TOF MS analysis also revealed that each lipopeptide family produced by *B. amyloliquefaciens* JFL21 was composed of different isoforms and fengycins exhibited variation in the length of the β-hydroxy-fatty acid from 14 to 19 carbons units (Figure 6B and Table 3). Our results are consistent with previous works reported by Jemil et al. (2017) and Perez et al. (2017) that showed the co-production of different homologous compounds of each lipopeptide family. However, the quantity of fengycin isoforms may be different in the various *Bacillus* species or isolates. For example, *B. subtilis* N6–34 could produce multiple fengycin homologs with chain lengths from C_15_ to C_17_ whereas *B. amyloliquefaciens* S13-3 only synthesize C_16_ fengycin variant (Yamamoto et al., 2015; Sa et al., 2018).

Except for the difference of the fatty acid chain length, fengycin isoforms also vary in the lactone rings of amino acid composition. According the previous literature, the variations at position 6 (Ala/Val), and variations at position 10 (Val/Ile) were mostly observed (Pathak et al., 2012; Cochrane and Vederas, 2016). Since the precursor ions mainly cleaved at the Glu–Orn and Orn–Tyr bonds, resulting in the specific octapeptide ring ions at *m/z* 952 and 1066 Da, 966 and 1080 Da, 980 and 1094 Da, and 994 and 1108 Da, respectively (Wang et al., 2004; Pathak et al., 2012; Yang et al., 2015; Sa et al., 2018). Moreover, the extra stability of the charged octapeptide ring system suppressed further fragmentation along the peptide backbone, and thus these specific product ions have been used as diagnostics for identifying fengycin variants with the amino acids replacement within the macrocyclic moiety (Pathak et al., 2012). Based on these specific product ions, the fengycin identified in the lipopeptides mixture Anti-JFL21 was readily assigned as four fenycin variants (fengycin A1, A2, B1, and B2) with chain lengths from C_13_ to C_19_, whereas the commercial fengycin standard was determined as two fenycin variants (fengycin A1 and B1) with lengths from C_13_ to C_18_. In addition, the MALDI-TOF MS/MS analysis revealed that a precursor ion of fengycin could be simultaneously identified as multiple fengycin variants, which indicated that it is a mixture of structural analogs and the further component purification are still required to obtain the mono variant or isoform.

Previously, fengycin was mainly characterized by strong antifungal activities against a wide range of plant pathogens, especially filamentous fungi (Guo et al., 2014; Fan et al., 2017; Zihalirwa Kulimushi et al., 2017). Nevertheless, it is particularly noteworthy that the fengycin produced by *B. amyloliquefaciens* JFL21 not only exhibited a remarkable antifungal activity comparable to the commercial fengycin standard but also showed stronger antibacterial efficacy against Gram-positive and Gram-negative foodborne pathogen as compared to the commercial fengycin standard (Table 4). This may be due to the commercial fengycin standard only contained two fengycin variants with the variations at position 6 (Ala/Val), whereas the fengycin identified in the present study was composed of four fengycin variants with the variations not only at position 6 (Ala/Val) but also at position 10 (Val/Ile) (Table 3). In addition, the various content combinations of homologs or isoforms may also lead to the different antimicrobial properties (Sabaté and Audisio, 2013; Kaur et al., 2017; Sa et al., 2018). However, to better clarify the structure-function relationships, the separation, characterization, and antimicrobial test of specific fengycin variants or isoforms are still highly necessary in the future study.

In general, an antimicrobial substance in a food item is by itself not likely to ensure complete safety, and therefore often used in combination with either other antimicrobial compounds, chemical reagents, or technologies such as ultraviolet sterilization, heat, and various pH pressure (Zacharof and Lovitt, 2012; Lee et al., 2016). The current study shows that the fengycins produced by *B. amyloliquefaciens* JFL21 not only maintained anti-*Listeria* activity over a broad pH and temperature range, but it was also active after treatment with ultraviolet sterilization, chemical reagents, and proteolytic enzymes (Table 5). The similar properties of highly thermostable and resistant to many proteolytic enzymes and a broad range of pH were also found in other *Bacillus*-derived antimicrobial CLPs such as surfactin (Sabaté and Audisio, 2013) and a lipopeptide mixture (Ben Ayed et al., 2014).

To date, the active mechanism of fengycins is less well known compared with iturins and surfactins. The possible mechanism for the antifungal activity of fengycins is that they interact with sterol and phospholipid molecules in membranes and thus alter the structure and permeability of the fungal cell membrane (Farzand et al., 2019; Hanif et al., 2019). However, the mechanism of interaction may vary and depend on the structural features of fengycins and pathogen species (Kaur et al., 2017; Sarwar et al., 2018). Recently, Piewngam et al. (2018) firstly revealed that β-OH-C_17_ fengycin B could potentially be used as quorum-sensing blockers to combat antibiotic-resistant *S. aureus*, which laid the foundation for probiotic *Bacillus* to comprehensively eradicate intestinal *S. aureus* colonization. Consequently, extensive studies are still required for fully understanding and characterizing the exact antimicrobial role of fengycins toward individual foodborne pathogen.

## Conclusion

In this study, three *Bacillus* strains with excellent antimicrobial properties named JFL21, LQG17, and LQG36, were isolated from food or seafood sources and identified closely related to the species of *B. amyloliquefaciens*, *B. subtilis*, and *B. halotolerans*, respectively. Besides, the model organism *B. subtilis* 168 was also exploited to compare the antimicrobial properties with the three *Bacillus* strains. The results showed that the capacity to produce antimicrobial substances varied greatly among different *Bacillus* species or isolates. Among them, the antimicrobial substances produced by *B. amyloliquefaciens* JFL21 was low toxicity to most probiotics but exhibited strong and extensive antimicrobial activities against multidrug-resistant foodborne pathogens, and thus have more application potential. Subsequently, the FITR, HPLC, and MALDI-TOF MS analysis revealed that the partially purified antimicrobial compounds, Anti-JFL21, was composed of multiple lipopeptides of the surfactin, fengycin, and iturin families. After the separation of different lipopeptide classes, the result of this work demonstrated that fengycins not only play a critical role in antimicrobial activity but also remain highly stable against a variety of enzymes, chemical reagents, and extreme conditions. To the best of our knowledge, this study is the first report that fengycin is a primary active compound of *B. amyloliquefaciens* against a broad range of foodborne pathogenic microorganisms including Gram-positive pathogens, Gram-negative pathogens, and fungal pathogens.

## Data Availability Statement

The original contributions presented in the study are included in the article/Supplementary Material, further inquiries can be directed to the corresponding author/s.

## Author Contributions

L-ZL, L-QG, and J-FL conceived and designed this experiment. L-ZL performed data analysis and was a major contributor in drafting the work. Q-WZ and TW helped revise the manuscript critically. L-ZL and Q-WZ carried out the antimicrobial experiment. L-ZL and Z-QZ performed the purification, characterization, and comparison of the antimicrobial metabolites from different *Bacillus*. HZ and C-FZ performed the isolation and purification of lipopeptides. TW and Q-YX performed the biochemical characterization of fengycin. All authors reviewed and approved the submitted manuscript to be published.

## Conflict of Interest

The authors declare that the research was conducted in the absence of any commercial or financial relationships that could be construed as a potential conflict of interest.

## Supplementary Material

Supplementary Figure 1 Reversed-phase HPLC analysis of the different fractions separated from the lipopeptides mixture Anti-JFL21. **(A)** fraction 16; **(B)** fraction 30; **(C)** fraction 55.

Supplementary Figure 2 Antimicrobial activity of different lipopeptide components separated from the lipopeptides mixture Anti-JFL21 by Sephadex LH-20 gel column chromatography. **(A–C)** *L. monocytogenes*; **(D–F)** *A. hydrophila*; **(G–I)** *C. gloeosporioides*; CK, 1 mg/mL Anti-JFL21; 13–18, the fractions from 11 to 18; 25–30, the fractions from 25 to 30; 55–60, the fractions from 55 to 60.
