# Supplementary material for: Isolation and Characterization of Fengycins Produced by Bacillus amyloliquefaciens JFL21 and Its Broad-Spectrum Antimicrobial Potential Against Multidrug-Resistant Foodborne Pathogens
Source: Front Microbiol. 2020 Dec 18;11:579621. doi: 10.3389/fmicb.2020.579621 (PMC7775374; doi:10.3389/fmicb.2020.579621)
Supplement: Supplementary Figure 1 — Reversed-phase HPLC analysis of the different fractions separated from the lipopeptides mixture Anti-JFL21. (A) fraction 16; (B) fraction 30; (C) fraction 55. [file Data_Sheet_1.PDF]

Supplementary Material

1 Supplementary Figures

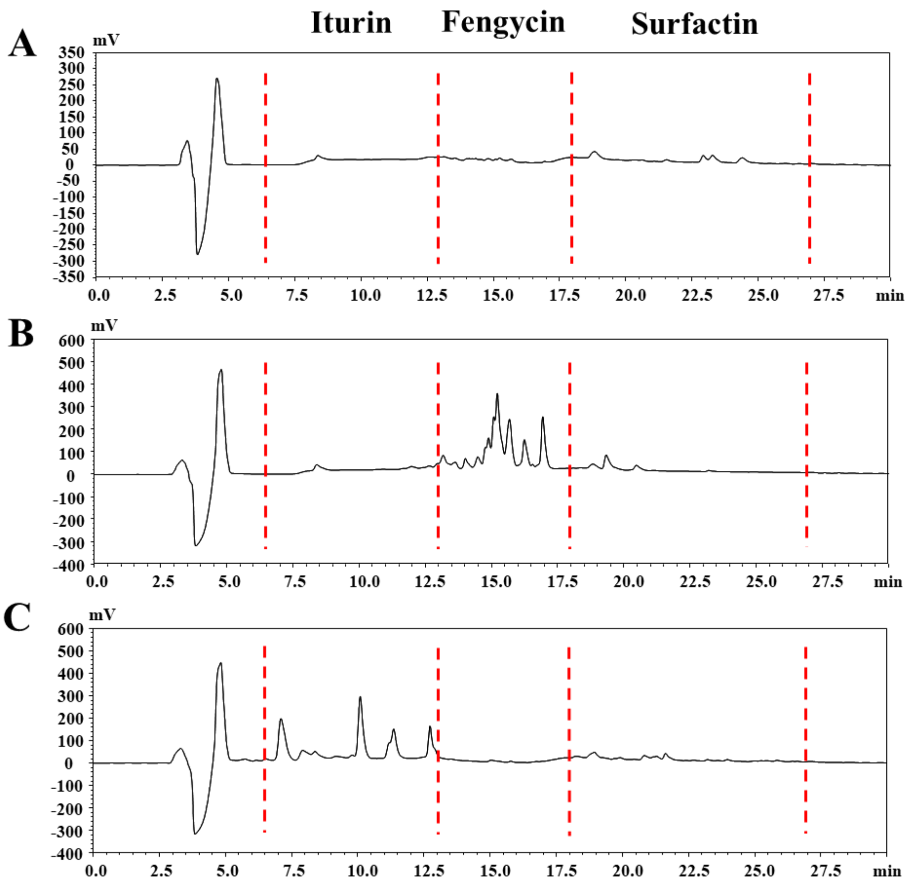

FIGURE S1 | Reversed phase HPLC analysis of the different fractions separated from the lipopeptides mixture Anti-JFL21. Only the representative fractions are given; **(A)** fraction 16; **(B)** fraction 30; **(C)** fraction 55.

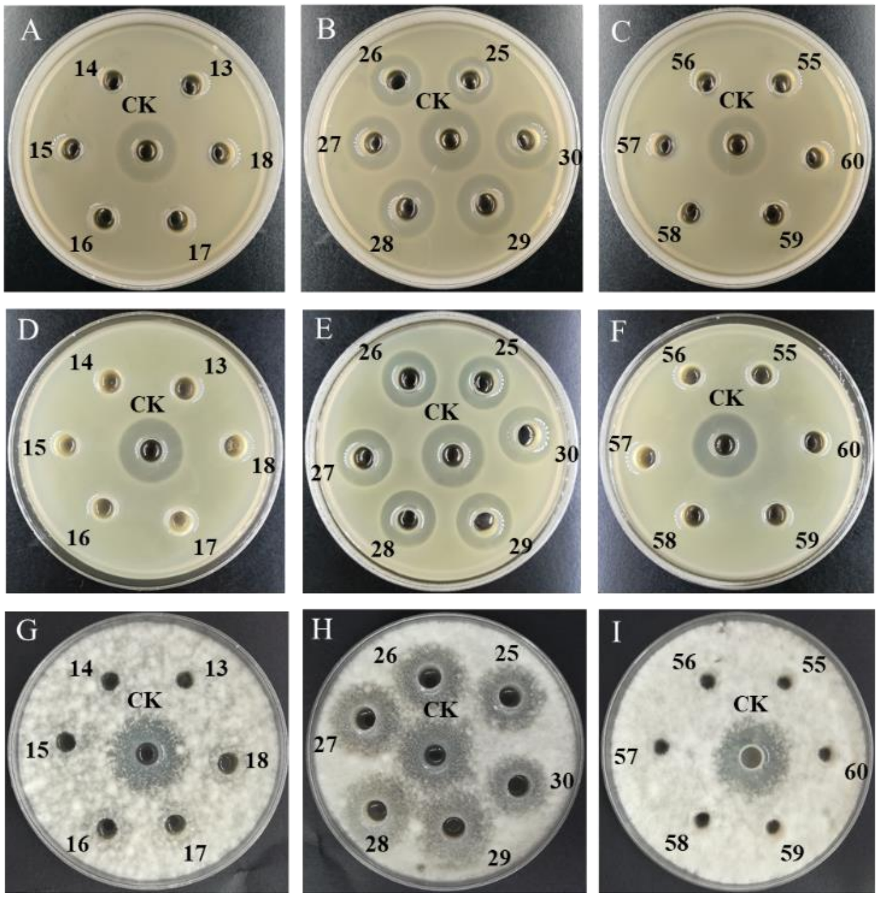

FIGURE S2 | Antimicrobial activity of different lipopeptide components separated from the lipopeptides mixture Anti-JFL21 by Sephadex LH-20 gel column chromatography. **(A-C)** *L. monocytogenes*; **(D-F)** *A. hydrophila*; **(G-I)** *C. gloeosporioides*; **CK**, 1 mg/ml Anti-JFL21; **13-18**, the fractions from 11 to 18; **25-30**, the fractions from 25 to 30; **55-60**, the fractions from 55 to 60.
